# spaGFM is a scalable graph foundation model for spatial transcriptomics analyses

**DOI:** 10.21203/rs.3.rs-11012643/v1

**Published:** 2026-09-18

**Authors:** Yu Zhong, Fei He, Xiaojie Jin, Yang Yu, Ricardo Melo Ferreira, Andrew J. Gunderson, Jordan E. Krull, Guangyu Wang, Michael T Eadon, Qin Ma, Dong Xu, Juexin Wang

**Affiliations:** 1. Department of Biomedical Engineering and Informatics, Luddy School of Informatics, Computing, and Engineering, Indiana University Indianapolis, Indianapolis, IN, USA; 2. Health Informatics Institute, University of South Florida, Tampa, FL, USA; 3. Department of Biomedical Informatics, College of Medicine, The Ohio State University, Columbus, OH, USA; 4. Department of Electrical Engineering and Computer Science, University of Missouri, Columbia, MO, USA; 5. Department of Medicine, Indiana University School of Medicine, Indianapolis, IN, USA; 6. Department of Surgery, The Ohio State University, Columbus, OH, USA; 7. Center for Bioinformatics and Computational Biology, Houston Methodist Research Institute, Houston, TX, USA; 8. Pelotonia Institute for Immuno-Oncology, The James Comprehensive Cancer Center, The Ohio State University, Columbus, OH, USA

## Abstract

Foundation models offer a promising paradigm for modeling spatial transcriptomics, but capturing tissue context over cellular graphs makes training at scale challenging. We introduce spaGFM, a graph foundation model that serializes cellular neighborhoods through random walks to generate transformer-compatible representations of tissue organization. By replacing classical graph message passing with augmented self-supervised neighborhood reconstruction, spaGFM captures higher-order spatial context while allowing scaling at the atlas level. Pretrained on 43.9 million cells from 132 image-based spatial transcriptomics datasets, spaGFM produces robust representations at both the neighborhood and cell level that transfer across tissues, disease contexts, and spatial technologies. SpaGFM identifies tertiary lymphoid structures across cancer cohorts and spatial platforms, captures cell-level transcriptomic perturbation responses associated with T cell vicinity in spatial CRISPR experiments, and characterizes glomerular organization associated with pathological grade in diabetic kidney disease biopsies. Overall, spaGFM establishes a graph foundation-model framework for learning cellular organization and its functional consequences.

A central question in biology is how diverse molecular states and cellular relationships collectively give rise to tissue organization, function, and disease. The rapid accumulation of single-cell and spatially resolved transcriptomics (SRT) provides an unprecedented opportunity to learn generalizable principles of cellular identity and state rather than to analyze each dataset in isolation. Single-cell foundation models such as scGPT^1^, scFoundation^2^, and GeneFormer^3^ have been further motivated by scaling laws first characterized in natural language processing, in which model performance improves predictably as the amounts of training data, computation, and model parameters increase^4^. However, high-resolution SRT measures cellular gene expression within intact tissue architecture. The quantified spatial relationships among cells are non-linear and graph-structured, creating a mismatch with sequence-based tokenization and complicating the direct application of transformer scaling strategies to SRT.

Current foundation models for SRT can be broadly categorized into expression-based and graph-based approaches. Expression-based models adapt pretraining paradigms for single-cell transcriptomics and incorporate spatial information during adaptation and applications. For example, Nicheformer^5^ learns representations from gene expression tokens augmented with spatial metadata, scGPT-spatial^6^ extends scGPT through continual pretraining with spatially-aware sampling and neighborhood reconstruction objectives. Graph-based methods instead represent tissue architecture explicitly as cellular graphs, with nodes corresponding to cells and edges encoding spatial proximity. CellTransformer^7^ and Novae^8^ leveraged graph neural networks (GNNs)^9^ to learn representations of local cellular neighborhoods. Compared with expression-based models, graph modeling explicitly assumes the local cellular neighborhood should share similar representations, thereby capturing multicellular organization that may be difficult to encode as auxiliary objectives. However, conventional GNNs rely on iterative message-passing mechanisms susceptible to over-smoothing, over-squashing, and difficulty representing long-range dependencies^10^, which complicate the joint scaling of receptive field and model capacity.

Here, we introduce spaGFM, a graph foundation model for spatial transcriptomics that scales relation model capacity with parameter size and sampled spatial context at the atlas level. spaGFM represents cell relations in SRT as cellular graphs but avoids the iterative message-passing bottleneck of GNNs by serializing local cellular neighborhoods through random walks and converting graph-structured spatial context into scalable transformer-compatible token sets. Graph structure determines which cells are co-sampled and augmented within each neighborhood, whereas a self-supervised reconstruction objective learns relationships among the sampled representations. We pretrained spaGFM on atlas-level SRT data to learn transferable representations of cellular and neighborhood organization.

We evaluated spaGFM across various spatial biological settings and at both cellular and neighborhood scales, and examined how representation quality scaled with model size, pretraining progress, and sampled spatial context. We further showed generalizability to unseen tissues and technologies, and assessed the scalability, transferability, and biological utility of spaGFM across diverse spatial biological research with SRT. With lightweight task-specific adaptation, spaGFM enables robust detection of tertiary lymphoid structures (TLSs) across multiple spatial transcriptomics platforms, characterization of cell-level transcriptomic perturbation responses associated with T cell vicinity, and accurate identification of glomeruli in diabetic kidney disease (DKD). As spatial datasets continuously grow in size, scalable models such as spaGFM that jointly represent molecular state and tissue organization will become increasingly important for linking cell atlases to mechanisms underlying tissue function and disease.

## Results

### Overview of spaGFM

**Fig. 1.**
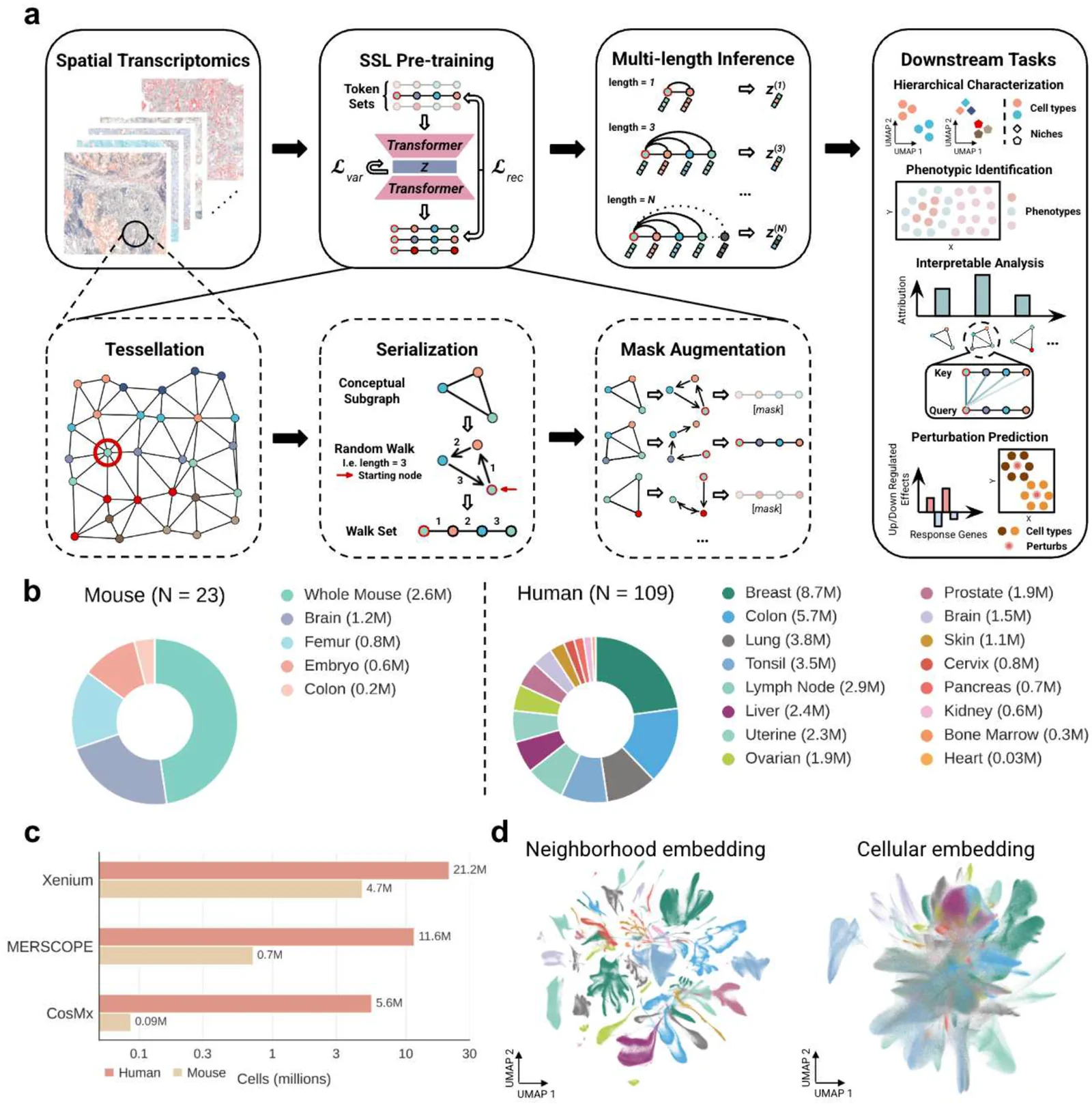
Schematics of spaGFM framework. (a) Overview of spaGFM model. spaGFM is pretrained on single-cell-resolution spatial transcriptomics data with self-supervised learning. Given the input spatial transcriptomics samples, cellular graphs are created by Delaunay Triangulation, and each graph is sampled into multiple neighborhood token sets using random walks. Each node represents a single cell characterized by its gene expression profile, with colors conceptually indicating cell types. spaGFM learns sampled neighborhood views by reconstructing masked walk sets with a Masked Autoencoder. This pretrained model supports multiple downstream applications. (b-c) The distribution of the pretraining datasets illustrated by tissue type and spatial transcriptomics platform. (d) UMAP of neighborhood-level and cell-level embeddings from the human pre-training datasets. For visualization, 10% of cells were subsampled from each dataset and colored by tissue type according to the color scheme in (b).

spaGFM is a scalable graph-based foundation model that learns cell organization representations from local spatial neighborhoods in SRT. Given a cellular-resolution SRT sample, spaGFM models the tissue as a cellular graph in which nodes encode cell-level gene expression profiles and edges are constructed from spatial coordinates using Delaunay triangulation^11^. The resulting graph is serialized into sets of neighborhood tokens by sampling local subgraphs through random walks (Fig. 1a). Inspired by previous works^12,13^, spaGFM is pretrained using a self-supervised masked autoencoder (MAE) objective that reconstructs masked neighborhood elements from observed context (Supplementary Fig. 1a). A variance regularization term is additionally adapted to reduce embedding collapse (Supplementary Fig. 2). In contrast to conventional GNNs, increasing the number and length of random walks augments the sampled spatial context without a performance bottleneck from deeper message-passing stack (Supplementary Fig. 3).

spaGFM is pretrained on an assembled large-scale corpus of image-based SRT data comprising three major platforms with cellular and subcellular resolutions, including 10X Xenium, CosMx SMI, and MERSCOPE (Methods). This corpus includes 43.9 million cells from 132 human and mouse samples spanning 20 tissues and diverse experimental conditions (Fig. 1b, c).

spaGFM learns hierarchical representations at both the cell and neighborhood scales within a unified framework. Cell-level representations support characterization of individual cells conditioned on their local context, whereas neighborhood-level representations summarize local tissue composition and organization (Supplementary Fig. 1b). In human samples, neighborhood representations preserved tissue-associated structure, whereas cell-level embeddings showed greater overlap across tissues (Fig. 1d). The pretrained representations can be used directly or adapted with lightweight task-specific models for prediction and spatial subgraph analyses (Fig. 1a). The framework can also incorporate gene-level prior knowledge for predicting spatial context-specific perturbation responses.

### spaGFM scales representation performance with model capacity and sampled neighborhood context

We benchmarked spaGFM’s hierarchical representations at both the cellular and neighborhood spatial scales in human and mouse datasets. Cell embeddings at the cellular level were evaluated for cell type prediction and neighborhood embeddings at neighborhood level were evaluated for niche identification, with comparisons to Novae, Nicheformer, and scGPT-spatial. To evaluate the quality of frozen representations directly from the spaGFM model, embeddings were extracted without task-specific encoder fine-tuning and assessed using linear probing, k-nearest neighbors (KNN) classification, and unsupervised clustering (Methods).

**Fig. 2.**
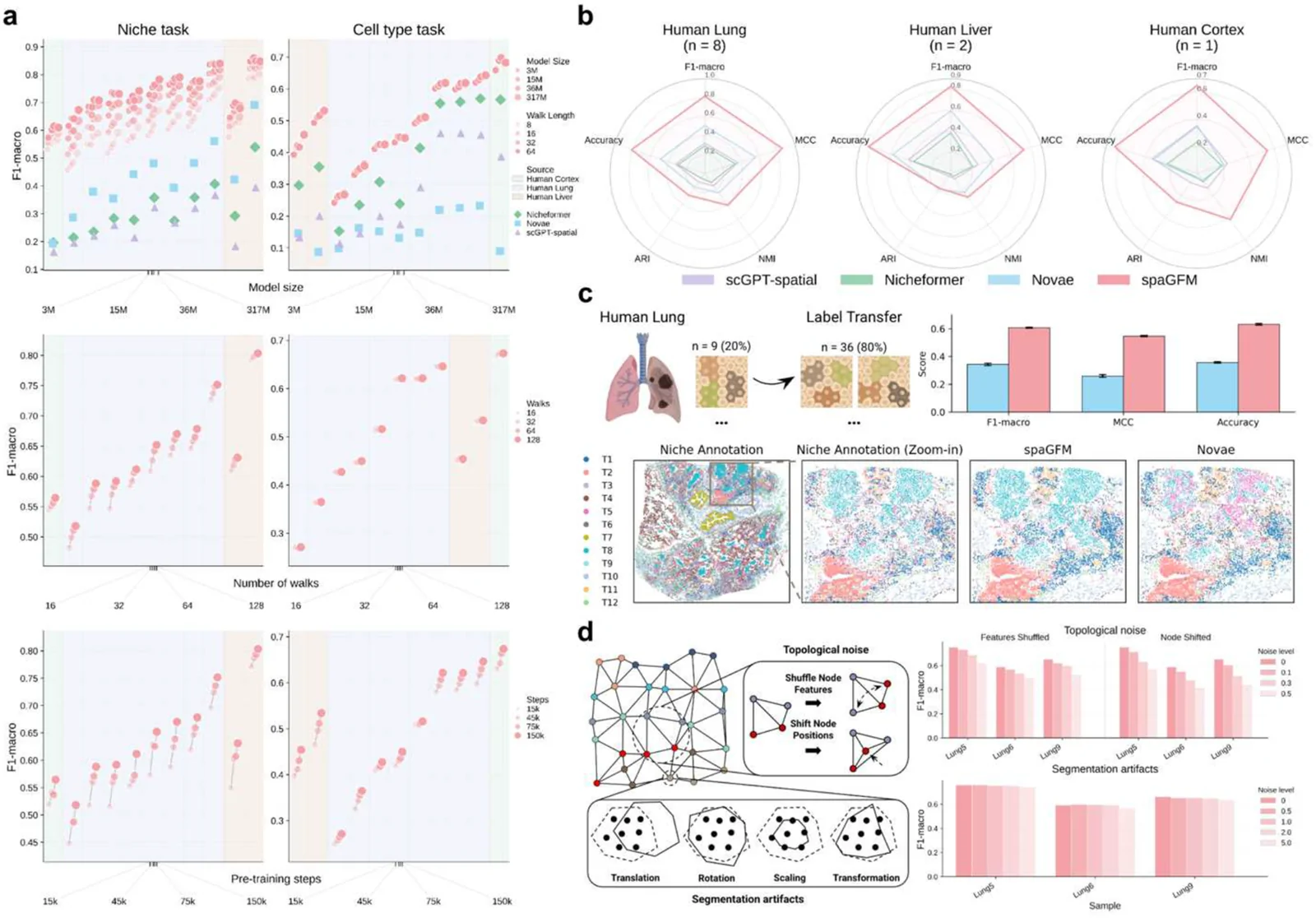
Evaluation of spaGFM on neighborhood and cell-level tasks. (a) Predictive performance of spaGFM as a function of model size (top): 3M, 15M, 36M and 317M; number of sampled random walks (middle): 16, 32, 64 and 128; and pretraining progress (bottom): 15k, 45k, 75k and 150k; across niche (left column) and cell type (right column) classification tasks. In linear-probing settings, three CosMx human tissues were used to compare spaGFM with baseline models, including Nicheformer, Novae, and scGPT-spatial. (b) Performance of niche tasks in multiple human tissues under KNN classification (F1-macro, Accuracy, and MCC; top) and K-means clustering (ARI and NMI; bottom), averaged across samples within each tissue. (c) Cross-patient evaluation of niche representations in human lung on the Xenium platform. Evaluation scheme for zero-shot niche-label transfer using embeddings derived from spaGFM or Novae in the human lung (top) datasets. Spatial visualization of niche predictions (bottom) for the representative sample across methods. (d) Schematic illustrations of topological-noise simulation (top left) and geometric boundary-perturbation simulation (bottom left) in the human lung with CosMx technologies. Under linear probing settings, performance was evaluated across varying levels of graph noise and geometric boundary perturbations (right). MCC: Matthews Correlation Coefficient. ARI: Adjusted Rand Index. NMI: Normalized Mutual Information.

Under linear probing, spaGFM performance increased with model size and pre-training progress across multiple tissues (Fig. 2a). Consistent with the hierarchical representation design, cell embeddings performed best for cell type classification, whereas neighborhood embeddings were most effective for niche identification (Supplementary Fig. 4a). Across the tested parameter range, performance improved up to the 317-million-parameter model. spaGFM also showed strong performance relative to the comparison methods under KNN classification and clustering across human (Fig. 2b) and mouse datasets (Supplementary Fig. 5). To assess transferability across individuals, we further evaluated niche label transfer in unseen human idiopathic pulmonary fibrosis (IPF) lung tissue^14^ and supratentorial ependymoma brain tissue^15^, where spaGFM outperformed Novae and supported more fine-grained niche annotation on both datasets (Fig. 2c, Supplementary Fig. 6).

Notably, niche identification was more dependent on graph context and walk-based augmentation than cell type classification (Fig. 2a), where increasing walk length and contextual coverage consistently improved niche characterization by providing a broader approximation of the local cellular environment. Across tissues, longer walks and more walks per cell provided robust niche-level characterization spanning a range of cellular densities (Supplementary Figs. 9a-b, 10a-b, 11a-b, 12a-b, 13). Tissues with higher cellular heterogeneity tended to require longer walks to characterize the niche than tissues with more homogeneous cell-type compositions (Supplementary Figs. 9c, 10c, 11c, 12c). These results suggest that neighborhood-level tasks benefit from higher-order spatial context and that the useful receptive field is tissue-dependent. Although spaGFM was pretrained with a fixed, relatively short walk length (Methods), variable walk lengths can be used at inference to adapt aggregation of spatial context without retraining.

To assess the modeling robustness^16^, we evaluated spaGFM under topological corruption and geometric perturbations (Fig. 2d; Methods). We observed that simulated node feature shuffling disrupted local cell-type composition, whereas coordinate perturbation distorted graph topology (Supplementary Fig. 14). The linear probing performance reasonably declined gradually as corruption increased, without an abrupt collapse. In approximating segmentation-related geometric perturbations^17^ with simulated translation, rotation, scaling, and shear of cell boundaries (Fig. 2d; Methods), neighborhood embeddings remained comparatively stable, supporting formulation robustness to this topological and geometric noise.

We next tested whether random-walk sampling and augmentation alone improved niche prediction. The neighborhood-level representations of the comparative baselines were constructed by max- or mean-pooling cell embeddings sampled along the same random walk without graph structure (Supplementary Fig. 7a). spaGFM consistently outperformed these pooled baselines across datasets (Supplementary Fig. 7b), indicating that random-walk sampling on the modelled cellular graph effectively improved niche prediction.

### spaGFM identifies Tertiary Lymphoid Structures across spatial transcriptomics platforms

Tertiary lymphoid structures (TLSs) are ectopic lymphoid aggregates that develop in non-lymphoid tissues at sites of chronic inflammation^18^, where their presence and maturation state are associated with prognosis, immunotherapy response, and disease progression across multiple cancers and inflammatory diseases. Despite their clinical importance, identifying TLS from SRT remains challenging. The widely used 10x Visium platform captures transcripts from multiple cells within each 55-μm spot, resulting in mixed cellular signals that obscure TLS boundaries. Furthermore, TLSs span a continuum of maturation states, from diffuse lymphoid aggregates to germinal center-like structures, limiting the effectiveness of marker-based approaches^19,20^.

We investigated spaGFM on previously unseen 10x Visium datasets. Because spaGFM was pretrained exclusively on higher-resolution imaging-based SRT datasets, transfer to spot-level Visium data provides a test of generalization across technologies and resolutions. To accommodate Visium profiles, we adapted scGPT to Visium data using scPEFT^21^ and used the resulting spot embeddings as input for spaGFM. spaGFM was fine-tuned to distinguish mature TLSs, early-stage TLSs, and non-TLS regions with expert annotations (Methods).

**Fig. 3.**
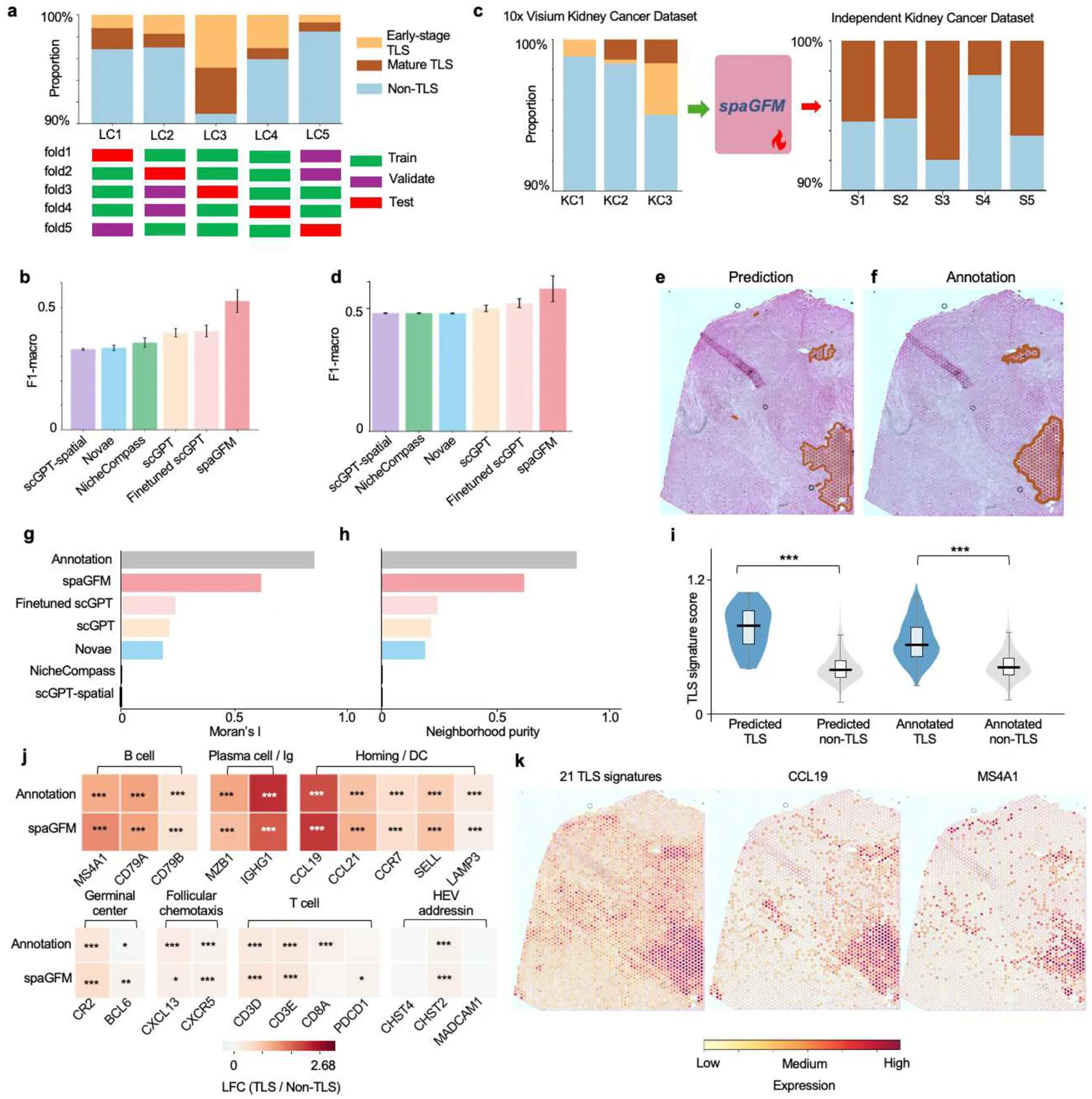
Benchmarking spaGFM for TLS identification across cancer types. (a) Cross-validation scheme and TLS annotation subsampling strategy on the 10x Visium lung cancer spatial transcriptomics dataset, with the proportion of annotated TLS spots. (b) F1-macro scores of spaGFM and comparison methods on the lung cancer dataset. (c) Workflow where spaGFM was fine-tuned on the 10x Visium kidney cancer dataset and evaluated on the independent renal cell carcinoma cohort (GSE175540). Bar plots show the proportions of TLS annotations in both datasets. The displayed y-axis begins at 90%. (d) F1-macro scores of spaGFM and comparison methods on GSE175540. (e) spaGFM-predicted TLS regions (shown in brown) overlaid on the H&E image of sample S1 from GSE175540. (f) Pathologist-annotated TLS regions (brown spots) overlaid on the same tissue section. (g) Moran’s I for pathologist annotations and predictions generated by spaGFM and comparison methods across the samples from GSE175540. (h) Neighborhood purity of pathologist annotations and model predictions across the same samples. (i) TLS signature score as mean log-normalized expression of 21 canonical TLS marker genes across TLS and non-TLS regions. Spot-level statistical comparisons were performed (two-sided Mann-Whitney U test with Bonferroni correction, all P < 10^−^^6^; ***). (j) Log2 fold-change in expression of 21 canonical TLS marker genes between TLS and non-TLS regions pooled across all five samples from the kidney cancer dataset, based on pathologist annotations (top) and spaGFM predictions (bottom). Genes are grouped by their associated TLS cellular or functional compartments. Colors indicate log2 fold-change (TLS/non-TLS). Statistical significance was assessed using two-sided Mann-Whitney U tests with Benjamini-Hochberg correction across the 21 genes (q < 0.05, q < 0.01, q < 0.001). (k) Spatial expression heatmaps of 21 TLS markers, CCL19, and MS4A1 overlaid on the H&E image. TLS: tertiary lymphoid structure.

We first evaluated spaGFM on a 10x Visium lung cancer dataset^22^. The sample containing the most abundant early-stage and mature TLS regions (LC3) was reserved as an independent test set, whereas the remaining four samples were used for four-fold cross-validation (Fig. 3a). We compared spaGFM with native scGPT, fine-tuned scGPT without spatial modeling, NicheCompass^23^, and the spatial foundation models Novae and scGPT-spatial. Across all validation folds in this pipeline-level comparison, spaGFM achieved the highest F1-macro score and consistently outperformed all competing methods (Fig. 3b). The improvement over fine-tuned scGPT, which provided the spot-level expression embeddings used by spaGFM, underscored the value of incorporating spatial context via graph-based random-walk tokenization and Transformer-based representation learning. Moreover, spaGFM consistently outperformed NicheCompass, Novae and scGPT-spatial, indicating that spatial representations learned from high-resolution imaging datasets transfer effectively to lower-resolution Visium data.

We next evaluated cross-dataset generalization using an independent renal cell carcinoma Visium dataset (GSE175540)^24^. spaGFM was fine-tuned using a 10x Visium kidney cancer dataset^22^ with three labels (mature TLS, early-stage TLS, and non-TLS) and directly applied to the kidney cancer dataset without additional retraining (Fig. 3c). Because the kidney cohort contains only binary TLS annotations, predictions of mature and early-stage TLSs were merged into a single TLS category for evaluation. Under this setting, NicheCompass and scGPT-spatial failed to identify TLS regions, and other comparison methods showed limited discriminative performance. In contrast, spaGFM consistently achieved the highest performance, improving F1-macro by approximately 10 percentage points relative to competing approaches (Fig. 3d).

spaGFM predicted TLS regions overlapped with pathologist-annotated TLSs on corresponding H&E images (Fig. 3e, f). Moran’s I and neighborhood purity analyses showed that spaGFM predictions were more spatially coherent than those of the comparison methods across the kidney cancer samples (Fig. 3g, h). We further assessed biological consistency using the expression of 21 canonical TLS marker genes (Methods). TLS signature scores^25^ (Mean log-normalized expression scores) were substantially higher in predicted TLS regions than in non-TLS regions (Fig. 3i), and predicted TLS regions recapitulated marker expression patterns observed in annotated TLSs (Fig. 3j, k). These results were further confirmed by spatial expression of *CCL19* and *MS4A1* colocalizing with spaGFM-predicted TLS regions (Fig. 3l), consistent with organized T-cell and B-cell compartments in TLSs^26^.

### spaGFM captures spatially conditioned perturbation responses

High-throughput genetic perturbation experiments, including Perturb-seq, are widely used to infer gene function from transcriptional responses at single-cell resolution. Perturb-FISH^27^ extends this paradigm to intact tissue by combining imaging-based SRT with *in situ* optical detection of guide RNAs, thereby capturing both cell-intrinsic perturbation responses and responses associated with the surrounding cellular microenvironment.

**Fig. 4.**
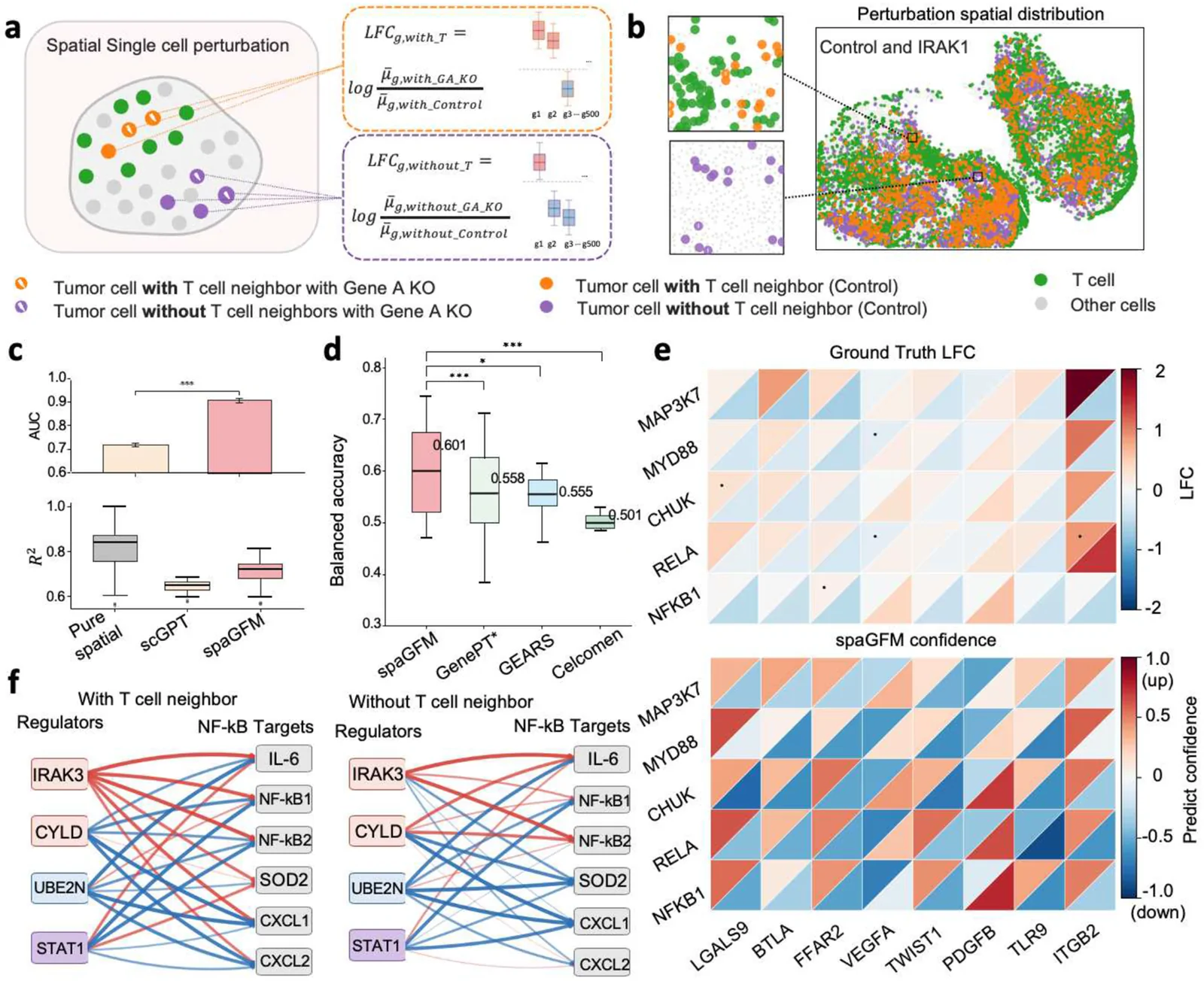
spaGFM predicts environment-dependent perturbation responses in spatial CRISPR (Perturb-FISH) tumor research. (a) Schematic of the prediction task. LFCs are calculated separately for tumor cells with and without T-cell neighbors. g1, …, g500 denote the 500 Perturb-FISH panel genes. Exemplary g2 illustrates a context-dependent change in LFC direction, whereas g1 and g3 illustrate changes in LFC magnitude but independent of spatial context. (b) Spatial distribution of IRAK1-knockout and control cells across the slide. Insets show a T-cell-proximal region (top) and a T-cell-distal region (bottom). (c) Validation and benchmarking. Top left, AUC for predicting T-cell proximity from the spaGFM embedding (red) versus an scGPT-based representation (yellow). Bottom left, R^2^ between the predicted LFC residual across methods (Pure spatial, scGPT, spaGFM) and the LFC provided in the original publication. (d) Prediction performance on held-out perturbations, shown as balanced accuracy for spaGFM, GenePT*, GEARS and Celcomen. Bars indicate the mean. GenePT* is the spatial-ablated control (Methods). *P < 0.05; **P < 0.01; ***P < 0.001. (e) Gene-level comparison of ground-truth and predicted effects for perturbed genes against target genes, colored by down-regulation (blue) to up-regulation (red). Top panel, ground-truth LFC. Bottom panel, spaGFM prediction confidence. Each upper-left triangle shows the target gene response to a perturbed gene in tumor cells with T cell neighbors, each lower-right triangle in tumor cells without T cell neighbors. Genes on the Y-axis represent perturbed genes, and genes on the X-axis represent responding genes. Dots mark entries with Wilcoxon Benjamini-Hochberg adjusted q < 0.1. (f) Predicted regulatory maps for canonical NF-κB pathway genes absent from the screen, linking upstream regulators to NF-κB targets in tumor cells with (left) and without (right) T cell neighbors. Targets represent inflammatory output (IL-6, CXCL1 and CXCL2), pathway feedback (NFKB1 and NFKB2) and cytoprotection (SOD2). Regulator nodes are colored by pathway annotation: negative regulator (red), positive transducer (blue), or interferon-pathway crosstalk component (purple). Target nodes are gray. Edge color denotes predicted up- (red) or down-regulation (blue) following regulator knockout, and edge width denotes confidence. LFC: log-fold change. AUC: area under the curve.

We applied spaGFM to a Perturb-FISH tumor study perturbing NF-κB genes to investigate context-dependent perturbation responses (Fig. 4a). Tumor cells carrying the same genetic perturbation occupied distinct spatial neighborhoods, allowing us to determine whether neighboring immune cells modulate transcriptional responses (Fig. 4b, Supplementary Fig. 15a).

Without explicit neighborhood annotations, spaGFM identified tumor cells with and without neighboring T cells in its learned embedding space (Supplementary Fig. 15b). This separation was quantitatively reflected by an AUC of 0.913 for predicting T-cell proximity from spaGFM embeddings, substantially exceeding the performance obtained using averaged scGPT embeddings of neighboring cells (AUC=0.721, Fig. 4c, top). This prediction power was further investigated to quantify spatial effects on perturbation responses. In leave-one-perturbation-out cross-validation across the 50 most variable genes, embeddings learned by spaGFM explained substantially more residual transcriptional variation than label-free neighborhood representations derived from averaged scGPT embeddings, approaching the performance of handcrafted spatial features that explicitly encode T-cell proximity (Fig. 4c, bottom). Further analysis revealed a small but statistically significant difference from the learned attention maps in the spatial space between T-cell-proximal and distal tumor cells (Cliff’s δ=−0.084, permutation P-value = 5×10^−4^, Benjamini-Hochberg-adjusted P-value = 1.4×10^−5^; Supplementary Fig. 15c, d), suggesting that the embedding contains information associated with T-cell proximity.

We next evaluated whether spatial representations improved prediction of unseen perturbations. spaGFM and GenePT^28^ could be evaluated on 70 held-out split-perturbation pairs, whereas GEARS^29^ and Celcomen^30^ supported only a subset of perturbations. For a strictly matched four-method comparison, we therefore restricted to the same 24 shared pairs (Supplementary Fig. 15e). The results show spaGFM significantly outperformed all comparison methods on this matched benchmark (Fig. 4d), where spaGFM specifically outperformed GenePT when retaining the full set of 70 pairs (Supplementary Fig. 15f).

At the individual gene level, spaGFM recovered the regulatory direction of selected transcriptional responses and assigned higher confidence to some larger perturbation effects. For example, perturbation of *MAP3K7*, an upstream activator of NF-κB signaling, produced the largest measured transcriptional changes that were reflected in model predictions (Fig. 4e). The model also predicted the correct direction for lower-magnitude effects, including the *CHUK*-*LGALS9*, *MYD88*-*VEGFA* and *RELA*-*ITGB2*, providing selected examples spanning a range of measured spatial effect sizes.

Finally, we investigated whether spaGFM could generate hypotheses for perturbations absent from the experimental screen. Canonical NF-κB regulators outside the Perturb-FISH panel were approximated by their nearest neighbors in GenePT embedding space, and then inferred their effects on canonical NF-κB targets^31^. The predicted results differed substantially between tumor cells with and without neighboring T cells (Fig. 4f), indicating candidate immune microenvironment-dependent perturbation effects. Many of the predicted outcomes were consistent with established functions of these regulators. For example, predicted loss of the negative regulators *IRAK3* and *CYLD* increased inflammatory target expression in T-cell-proximal cells, consistent with prior reports^32,33^. In contrast, the predicted loss of the positive regulator *UBE2N*, a K63 ubiquitin-conjugating enzyme required for TAK1 and IKK activation, reduced the same targets^34^. *STAT1* showed context-dependent predicted effects, including differential regulation of *CXCL2* between T-cell-proximal and distal tumor cells, consistent with context-dependent interactions between interferon-STAT1 and NF-κB signaling^35,36^. These zero-shot predictions represent hypotheses for targeted validation rather than measured effects, thereby extending them to biologically plausible regulatory interactions beyond experimentally assayed perturbations.

### spaGFM characterizes glomerular organization associated with pathological grade in diabetic kidney disease

**Fig. 5.**
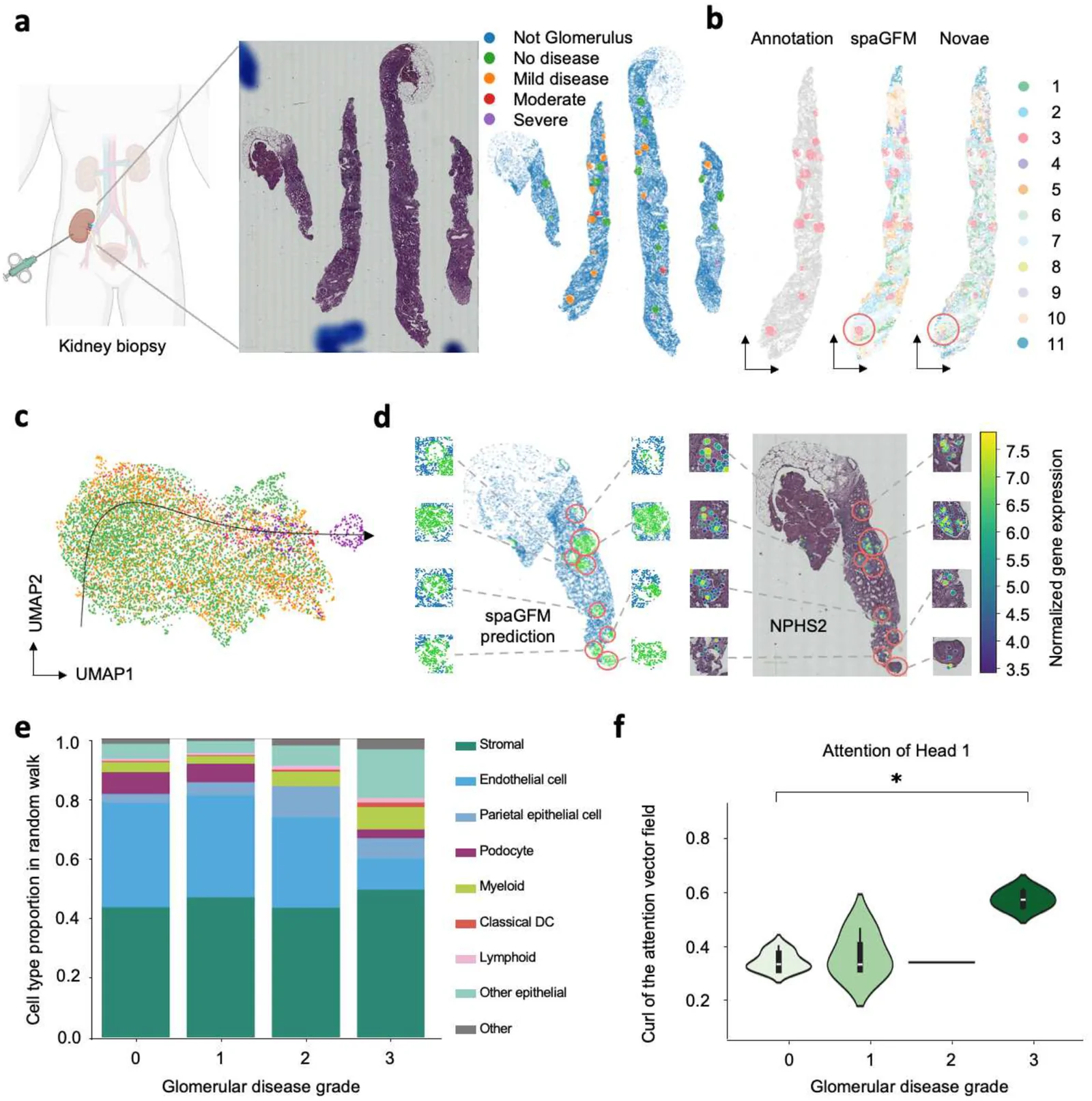
spaGFM resolves glomerular structure and pathological heterogeneity in diabetic kidney disease. (a) H&E-stained kidney biopsy from a diabetic kidney disease patient with 10X Xenium spatial transcriptomics shown alongside the spatial map of annotations by pathologists. Glomerular cells are colored by annotated pathological grade (Grade 0, no evident glomerular pathology; Grade 1, mild; Grade 2, moderate; Grade 3, severe), with non-glomerular cells labeled −1. (b) Spatial domains identified by manual annotation (left, red denotes glomerular cells, whereas gray denotes all other tissue), spaGFM (middle), and Novae (right) on the same kidney tissue section. Each cell is colored by its assigned domain label. Red circles exemplify the case where spaGFM identifies a more accurate glomerular structure than Novae. Arrows denote sample spatial orientation. (c) UMAP of glomerular cell embeddings with pseudo-time trajectory overlaid by Slingshot. Cells are colored by pathological grade (Grades 0–3); the inferred trajectory terminates in a region enriched for Grade 3 cells and is interpreted as a grade-associated ordering rather than longitudinal progression. (d) Glomerulus detection by the fine-tuned model. spaGFM model prediction (left; blue = none, green = predicted glomerulus, red circles highlight predicted glomerular regions), and spatial high expression of the podocyte marker NPHS2 (right) providing molecular support for the additional candidate regions. (e) Random-walk-derived cell-type composition for each glomerular grade. (f) Violin plot of curl of the Head 1 attention field across grades (0–3); higher curl in Grade 3 indicates a difference in the geometry of the inferred attention field (pairwise significance: two-sided Mann-Whitney U test, P-value< 0.05, *). The number of glomeruli across grades: 8 (grade 0); 3 (grade 1); 1 (grade 2); 3 (grade 3).

The kidney has a highly organized tissue architecture, and accurate identification of disease-associated glomeruli, a principal functional tissue unit, remains challenging in computational pathology and SRT analyses because the underlying glomerular cell types are significantly altered in expression and morphology by disease^37,38^. We analyzed four SRT cores (Splits 1–4) from a diabetic kidney disease (DKD) patient biopsy^39^. Cell identities and glomerular boundaries were manually annotated, and renal pathologists assigned each glomerulus to one of four pathological grades based on H&E morphology: no evident glomerular pathology (Grade 0), mild (Grade 1), moderate (Grade 2), and severe nodular mesangial sclerosis (Grade 3) (Fig. 5a, Supplementary Fig. 16a).

We first clustered zero-shot neighborhood embeddings and observed that the resulting spatial domains delineated glomerular structures. spaGFM partitioned the tissue into 11 spatial domains, with domain 2 closely corresponding to the manually annotated glomeruli. In comparison with Novae, the spaGFM-derived glomerular regions indicated greater structural continuity (Fig. 5b, Supplementary Fig. 16b-e). Trajectory analysis of neighborhood embedding revealed a continuous severity-associated ordering of glomerular cells across the four annotated categories (Fig. 5c), indicating the learned representations captured variation associated with pathological grade.

We further fine-tuned the spaGFM for per-cell glomerulus identification using 13 annotated glomeruli from Split 2 for training, five glomeruli from Split 4 for validation, and the remaining two sections for testing. spaGFM achieved greater than 90% accuracy and approximately 0.97 recall (Supplementary Fig. 16f). These predicted glomerular regions were confirmed with the expression of the podocyte marker gene *NPHS2*^40^ (Fig. 5d). When pathological grades were collapsed into Grade 0 and Grades 1–3 categories, the fine-tuned model maintained approximately 90% accuracy (Supplementary Fig. 16h). Notably, spaGFM performance scales with model sizes in this glomeruli-identification task. We compared the results between a reduced model of 36-million-parameters and the full model with 317-million-parameters and observed a performance gain in the representation benchmarks, including elimination of mis-annotations due to technical artifacts at the edge of the tissue (Supplementary Fig. 16).

To investigate the biological basis of the predictions, we analyzed the neighborhood composition captured along the augmented random paths in spaGFM. Neighborhood cell proportions showed grade-associated changes consistent with known DKD pathology (Fig. 5e). In particular, the relative loss of podocytes was consistent with disruption of the glomerular filtration barrier and podocytopathy frequently observed in advanced DKD. In contrast, expansion of the kidney stromal cells and parietal epithelial cells is consistent with mesangial expansion and pericapsular fibrosis, respectively, in diabetic kidney disease. Finally, myeloid and other immune cell populations were enriched in glomeruli with advanced disease.

Outside of Glomeruli, spaGFM was able to localize disease-state niches more precisely than Novae. For example, niche 7 corresponds to a fibro-immune niche (Supplementary Fig. 17). spaGFM distinctly maps niche 7 to its appropriate underlying histology (Supplementary Fig. 17a). Compared to a healthy proximal tubular niche (niche 2), we observed a corresponding fibrosis signature in niche 7 (Supplementary Fig. 17b, c). Novae was unable to precisely map niche 7 as these were broadly distributed and mixed among other niches.

We additionally examined the learned attention patterns as a potential source of model interpretation. Attention Head 1 emphasized Grade 3 cells, with high-attention regions enriched for pathological severity (Supplementary Fig. 18). We represented attention in physical space as a vector field and summarized its local geometric organization using curl and divergence. These quantities statistically differed in Grade 3 glomeruli (Fig. 5f) related to patterns across additional multiple attention heads (Supplementary Fig. 19a, b), which provides descriptive associations between attention geometry and pathological grade.

## Discussion

Rapidly accumulated atlas-level SRT data brought both challenges and opportunities in data modeling. spaGFM establishes a scalable graph foundation-model framework by converting spatial cellular graphs into random-walk neighborhood token sets that can be processed by transformer architectures. Compared to the conventional approaches (Supplementary Table 1), the central design separates the size of the sampled spatial context from the depth of graph message passing: local graph structure determines which cells are co-sampled, while self-supervised transformer learning models relationships among the sampled representations. Across the comprehensive experiments on different tissues, representation performance increased with model size, pretraining progress, and sampled neighborhood context. These observations support the scalability of the framework over the tested ranges, although we tested up to 317-million-parameters due to computational availability. A formal scaling-law analysis across data, parameters, and compute remains an important direction for future work.

Generalizability is a central characteristic of a foundation model. spaGFM uses Delaunay triangulation to construct spatial cellular graphs^11^, providing a consistent geometry-based inductive bias applicable across SRT datasets represented in two-dimensional Euclidean coordinates. This setting provides transferability in the modeled triangle-composed planar spatial cellular graphs under uniform Euclidean geometries^41^, which enables high-quality zero-shot learning and label transfer in diverse scenarios in SRT data.

The attention-vector-field analysis provides a complementary way to interpret spatial patterns learned by spaGFM. Visualizing learnt attention in physical space enables descriptive summaries of local orientation, curl, and divergence. In the DKD case study, these quantities were identified to be associated with pathological grade. Because attention weights are model-derived and are not equivalent to causal explanations, these results should be interpreted as exploratory measures of representation geometry rather than direct evidence of biological mechanisms.

A key feature of spaGFM is its ability to learn hierarchical representations across cellular and tissue-neighborhood scales. Cell-level embeddings capture information relevant to cell-state characterization, whereas neighborhood-level embeddings integrate local tissue composition and spatial organization, providing representations suited to niche-level analyses. Benchmarking and ablation experiments (Supplementary Table 2–4) further indicate that higher-order spatial context is particularly important for neighborhood-level tasks (Supplementary Fig. 7), with flexible random-walk sampling allowing the effective receptive field to be adjusted at inference without retraining. This flexibility is accompanied by favorable computational scaling: across the tested range, inference time increased approximately linearly with cell number, with limited sensitivity to tissue density or spatial architecture (Supplementary Fig. 4b). Although spaGFM can therefore be directly applied across datasets with distinct spatial characteristics, lightweight adaptation to previously unseen datasets can further improve predictive accuracy (Supplementary Fig. 8), highlighting a practical balance between generalizability and dataset-specific refinement.

The downstream applications illustrate the versatility of these pretrained representations. In cancer datasets, spaGFM was transferred from high-resolution imaging platforms to 10x Genomics Visium data, identifying TLS-associated regions across lung and renal cancer cohorts. In Perturb-FISH data, spaGFM embeddings captured spatial context associated with T-cell proximity and enabled prediction of held-out perturbation responses. Predictions for unmeasured NF-κB regulators extend this analysis to hypothesis generation, may facilitate prioritization of context-dependent perturbations for future Perturb-FISH, spatial CRISPR, and targeted validation studies. In the DKD study, zero-shot embedding and lightweight fine-tuning supported glomerulus identification and captured grade-associated spatial organization, while additional NPHS2-positive candidate regions suggested that molecular and spatial representations can complement morphology-based annotation.

There are still several limitations. First, spaGFM currently represents gene expression using pretrained cell embeddings rather than gene-level tokens, which limits direct modeling of gene-level regulatory programs and results in missing transcripts. Joint modeling of gene-level and neighborhood-context representations could connect intracellular regulation and intercellular organization. Second, spaGFM relies on predefined spatial graphs and random-walk sampling, therefore it depends on assumptions about tissue connectivity and spatial scale. Adaptive graph construction, histology-informed connectivity, and learned receptive fields may improve modeling of more complex tissue architectures. Third, although spaGFM was pretrained across multiple imaging-based SRT technologies and transferred to independent tissues and Visium datasets, broader validation across additional patients, sequencing-based and multimodal spatial assays, and three-dimensional tissues will be important for defining its generalizability. Finally, the DKD analysis derives from one patient, and the unmeasured perturbation predictions are not experimentally validated, which should therefore be regarded as proof-of-concept and hypothesis-generating analyses, respectively.

## Methods

### Spatial cellular graph construction

spaGFM models the SRT slice i with single-cell resolution as a graph $G_{i}=(V,E)$, where V is the set of cells as nodes and E as edges connecting each cell tessellated by Delaunay triangulation^11^. For each cell j, its two-dimensional spatial coordinates are denoted as $c_{j}$. To alleviate unreasonable connections between distant cells, suspicious edge connections are filtered by a distance threshold of spatial coordinates. The processed $G_{i}=\left(V,\left\{(u,v)\in E|{\left\|c_{u}-c_{v}\right\|}_{2}<q_{0.99}\right\}\right)$, where $q_{0.99}$ is 99^th^ quantile of the edge distance distribution for slice i. To harmonize gene panels across datasets, we initialized node features using the frozen scGPT^1^ whole-human pretrained checkpoint. For both human and mouse datasets, genes were matched to the scGPT vocabulary by gene symbol, and only genes with matching symbols were used to generate the D-dimensional scGPT cell embeddings. Together, the full unweighted graph representation for slice i with cell organization information is denoted as $G_{i}=\left(V,\left\{(u,v)\in E|{\left\|c_{u}-c_{v}\right\|}_{2}<q_{0.99}\right\},X\right)$ where $X\in R^{|V|\times D}$.

### Random-walk tokenization

The cellular graph of the slice i is further sampled into sets of neighborhood nodes by using a random-walk sampler. From each source node j, the walk sampler continuously travels through the graph $G_{i}$ according to its adjacency, generating a random walk $r_{ij}=\left(v_{i,j}^{0},v_{i,j}^{1},\ldots ,v_{i,j}^{L}\right)$ with length L, and $v_{i,j}^{l}\in V_{i}$. We used the idea from Node2Vec^42^, balancing the walking process between Breadth-First Search (BFS) and Depth-First Search (DFS). We denote $a_{t}$ as the $t^{\text{th}}$ node in the walk. Each step of the walk is sampled in Eq. (1), where $\pi_{vu}$ is the transition probability matrix between nodes u and v with Z as a normalized constant. The transition probability is determined by the shortest path distance denoted by d, between the $(t-2{)}^{\text{th}}$ node s and the $t^{\text{th}}$ node u, where d(s,u)∈{0,1,2}.


(1) $$P\left(a_{t}=u|a_{t-1}=v\right)=\left\{\begin{array}{ll} \frac{\pi_{vu}}{z} & \text{if}\;(u,v)\in E\\ 0 & \text{otherwise} \end{array}\right.$$

$$\pi_{vu}=\alpha_{su}w_{vu};\alpha_{su}=\left\{\begin{array}{ll} \frac{1}{p} & \text{if}\;d(s,u)=0\\ \frac{1}{q} & \text{if}\;d(s,u)=2\\ 1 & \text{if}\;d(s,u)=1 \end{array}\right.$$

Here we parameterized p=q=1, and $w_{vu}$ set as 1 as SRT is represented as unweighted graphs. This random-walk sampling was repeated and augmented N times independently for each node j in slice i to enrich the neighborhood representation, denoted as $R_{ij}={\left\{r_{ij}^{n}\right\}}_{n=1,\ldots N}$, where the $n^{\text{th}}$ walk $r_{ij}^{n}$ constitutes L nodes generated during random walk sampling.

Given the pair representation of node features and its neighborhood $\left(x_{ij},R_{ij}\right)$ of source node j in slice i, we tokenized the $n^{\text{th}}$ sampled neighborhood through transformer-based encoding^12^, denoted as $h_{ij}^{n}=f_{\theta }\left(x_{ij}^{n}\right)$, where $h_{ij}^{n}=\left(h_{iv_{i,j}^{\left(n,0\right)}},h_{iv_{i,j}^{\left(n,1\right)}},\ldots ,h_{iv_{i,j}^{\left(n,L\right)}}\right)$ and $x_{ij}^{n}=\left(x_{iv_{i,j}^{\left(n,0\right)}},x_{iv_{i,j}^{\left(n,1\right)}},\ldots ,x_{iv_{i,j}^{\left(n,L\right)}}\right)$. For the $n^{\text{th}}$ sampled neighborhood, each token encodes the $l^{\text{th}}$ node with features $x_{iv_{i,j}^{\left(n,l\right)}}$, and projects into high dimension representation $h_{iv_{i,j}^{\left(n,l\right)}}$ by transformer encoder^43^ $f_{\theta }$, across (L+1) steps. Spatial-aware cell embedding for the source node j represents as $h_{iv_{i,j}^{\left(n,0\right)}}$. The trainable tokenization per walk denoted as $\bar{h_{ij}^{n}}$ was averaged across steps via mean pooling in Eq. (2).


(2) $$\bar{h_{ij}^{n}}=\frac{1}{L+1}\sum_{l=0}^{L}h_{iv_{i,j}^{\left(n,l\right)}}$$

### Self-supervised pretraining for spaGFM

spaGFM uses a masked autoencoder (MAE) framework^44^ to learn the semantic representation of each cellular neighborhood in a self-supervised learning formulation (Supplementary Fig. 1a). Encoder $f_{\varphi }(\cdot )$ encodes the semantic relationship denoted as ${\left\{z_{ij}^{k}\right\}}_{k=1,\ldots K}$, between K visible walks, where ${\left\{z_{ij}^{k}\right\}}_{k=1,\ldots K}=f_{\varphi }\left({\left\{\bar{h_{ij}^{k}}\right\}}_{k=1,\ldots K}\right)$. Decoder $f_{\phi }(\cdot )$ reconstructs M masked walk representations, denoted as $\{y_{iJ}^{m}\hat{{\}}_{m=1,\ldots M}}$, out of total N walks, conditional on K visible walks along with masked tokens in Eq. (3).

(3) $$\{y_{lȷ}^{m}\hat{{\}}_{m=1,\ldots M}}=f_{\phi }\left({\left\{z_{ij}^{k}\right\}}_{k=1,\ldots K},[\text{MASK}]\right)$$

$f_{\varphi }(\cdot )$ and $f_{\phi }(\cdot )$ are standard transformer models parameterized by φ and ϕ, respectively^43^. In our setting, K=16 and N=128. This formulation is conceptually analogous to masked patch representation learning in computer vision^44^. We do not employ positional encoding either within or across walks. Consequently, each sampled walk is treated as a permutation-invariant multiset of neighborhood tokens rather than as an order-sensitive trajectory.

Because ground-truth walk representations are unavailable, we adopted an online-target learning strategy for reconstruction optimization^13,45^. The online targets ${\left\{y_{ij}^{m}\right\}}_{n=1,\ldots M}$ were constructed by the same encoder structure with gradient propagation stopped, denoted as sg(), during the training process in Eq. (4).


(4) $${\left\{y_{ij}^{m}\right\}}_{m=1,\ldots M}=\text{sg}\left(f_{\varphi^{\prime }}\left({\left\{\bar{h_{ij}^{\prime m}}\right\}}_{m=1,\ldots M}\right)\right)$$

$$\bar{h_{ij}^{\prime m}}=\frac{1}{L}\sum_{l=0}^{L}sg\left(f_{\theta^{\prime }}\left(x_{ij}^{m}\right)\right)$$

Only the target (teacher) parameters in $f_{\varphi^{\prime }}$ and $f_{\theta^{\prime }}$ were updated by Exponential Moving Average (EMA) rules in Eq. (5). We updated every 10 optimization steps with α=0.99. The online (student) encoder and decoder were optimized by backpropagation and received the reconstruction- and variance-loss gradients.


(5) $$\theta^{\prime }=\alpha \theta^{\prime }+(1-\alpha )\theta ;\varphi^{\prime }=\alpha \varphi^{\prime }+(1-\alpha )\varphi$$

The reconstruction objective is to minimize mean-squared error over masked positions in Eq. (6).


(6) $${ℒ}_{rec}=\frac{1}{\left|M\right|}\sum_{m\in M}{\left\|y_{ij}^{m}-\hat{y_{lj}^{m}}\right\|}_{2}$$

To prevent the variance of each embedding dimension from collapsing to a trivial solution, we added a penalty loss over the latent neighborhood representation $\bar{z_{ij}}$ to prevent its variance across the batch dimension below a threshold^46^ in Eq. (7). We define $\bar{z_{ij}}=\frac{1}{K}\sum_{k=1}^{K}z_{ij}^{k}$, which is the mean-pooled representation over the K visible walks. $S(x,\epsilon )=\sqrt{Var(x)+\epsilon }$, where γ is the target threshold of standard deviation and ϵ is a small scalar for numerical stability. Here, we used γ=1 with $\epsilon =1\times 10^{-4}$ as the default value.


(7) $${ℒ}_{var}=\frac{1}{D}\sum_{d=1}^{D}max\left(0,\gamma -S\left(\bar{z_{ij}},\epsilon \right)\right)$$

Together, the overall objective loss is defined as Eq. (8), the sum of reconstruction loss in Eq. (6) and variance loss in Eq. (7).


(8) $${ℒ}_{\text{total}}={ℒ}_{\text{rec}}+{ℒ}_{\text{var}}$$

Additional methodology details can refer to Supplementary Fig. 1.

### Cellular and Neighborhood representations in spaGFM

Cellular and neighborhood levels of cellular organization differ in granularity and impose distinct representational requirements within a unified embedding space. spaGFM addresses this challenge by learning representations at multiple levels during pre-training (Supplementary Fig. 1b). During inference, for a cell j in slice i, we define neighborhood embedding as $\bar{z_{ij}}=\frac{1}{N}\sum_{n=1}^{N}z_{ij}^{n}$ which performs mean aggregation over N walks for neighborhood-level characterization. For cell-level tasks, we use the cell embedding $\bar{h_{ij}}=\frac{1}{N}\sum_{n=1}^{N}h_{iv_{i,j}^{\left(n,0\right)}}$. Cell embeddings capture the source cellular representations while conditioning on the local spatial neighborhood, enabling cell-level characterization within its spatial context. In contrast, neighborhood-level embeddings aggregate information from the surrounding cellular environment, capturing both the composition and spatial organization of the local microenvironment for neighborhood-level analyses.

### The implementation of model pretraining

We developed a customized data loader to efficiently process large-scale spatial transcriptomics datasets during pretraining. Each slice was treated as an individual spatial cellular graph, and a mini-batch of nodes were selected across all graphs. In each training step, a batch of 256 cells was randomly sampled across all tissue slices. To avoid sampling bias toward larger graphs, nodes from smaller graphs were repeatedly resampled once exhausted during training. Models were pretrained using mixed-precision training (BF16) with the AdamW optimizer and a learning rate of 1 × 10^−4^). We employed a warmup-stable-decay scheduler with 3% warm-up steps and 90% stable steps. Human and Mouse models were pretrained independently. Training was performed on four NVIDIA H100 GPUs for 150k steps for the Human model and 100k steps for the Mouse model.

### Model scaling with parameter sizes

spaGFM scales model parameters primarily by increasing the width and depth of the transformer encoder (Supplementary Fig. 1c), denoted as $f_{\varphi }(\cdot )$, and the transformer decoder, denoted as $f_{\phi }(\cdot )$. The encoder and decoder widths are denoted by $d_{\text{model}}$, corresponding to the hidden dimension of each transformer block, where $d_{\text{model}}\in \{256,640,768,1280\}$. The encoder and decoder depths are denoted by $L_{\text{encoder}}$ and $L_{\text{decoder}}$, respectively, corresponding to the number of transformer layers, where $L_{\text{encoder}}\in \{1,2,3,12\}$ and $L_{\text{decoder}}\in \{1,1,1,3\}$. We use a fixed attention head dimension of 64 for all models, with the number of heads $H=d_{\text{model}}/64$, and feedforward width $d_{ff}=4d_{\text{model}}$. We trained four spaGFM variants with different hidden dimensions and numbers of transformer layers, resulting in spaGFM-3M, spaGFM-15M, spaGFM-36M, and spaGFM-317M with 3, 15, 36, and 317 million parameters, respectively. Notably, we use full-size spaGFM-317M in all the case studies.

### Ablation tests in model and applications

To validate the contribution of each computational component of spaGFM, we performed sophisticated ablation analysis of the spaGFM model, including initial representation, graph representation, transformer backbone, and variance regularization was summarized in Supplementary Table 2. The ablation analysis in applications was summarized in Supplementary Tables 3 and 4.

### Data collection and preprocessing

#### Pretrained datasets for spaGFM

Pre-training spatial transcriptomics datasets were collected and downloaded from their official depositories (Data Availability). We filtered out cells with fewer than 10 expressed genes and genes detected in fewer than 5 cells. Expression counts were normalized by the median cell-by-gene count depth and log1p-transformed. We selected the CosMx Human Lung, Liver, and Cortex datasets and the CosMx Mouse Brain dataset for downstream evaluation because they include annotated cell-type and niche labels. These datasets were excluded from the pretraining corpus and used only for downstream evaluation.

#### Human idiopathic pulmonary fibrosis (IPF) lung and supratentorial ependymoma brain in 10x Xenium spatial transcriptomics

Human lung (GSE250346^14^) and brain (GSE300146^15^) datasets were obtained from the GEO database. We directly used their pre-processed datasets without post-processing steps.

#### Tertiary Lymphoid Structures studies in 10x Visium spatial transcriptomics

We evaluated spaGFM using a 10x Genomics Visium spatial transcriptomics dataset^22^ comprising lung and kidney tumors with diverse tertiary lymphoid structure (TLS) architectures. The dataset includes three kidney cancer samples (KC1-KC3) and five lung cancer samples (LC1-LC5) derived from FFPE tumor sections. Tissue sections (5 μm) were profiled using the Visium Spatial Gene Expression platform with the Human Probe Set v1, followed by H&E staining, library preparation, and sequencing on an Illumina NovaSeq 6000. Spatial transcriptomic data were processed with Space Ranger v2.1.0, and Visium spots were manually annotated by expert pathologists as mature TLS, early TLS, immune region, tumor region, normal tissue, or unlabeled. Unlabeled spots were excluded from downstream analyses. This dataset was used to assess the ability of spaGFM to identify TLS-associated microenvironments across multiple cancer types and TLS developmental states.

To evaluate cross-dataset generalization, we further analyzed an independent renal cell carcinoma spatial transcriptomics dataset^24^. This dataset comprises five tumor samples generated from both FFPE and fresh-frozen tissues and sequenced on the Illumina NovaSeq 6000 platform. Spatial regions were annotated as TLS-associated or non-TLS-associated niches. We used this cohort to assess whether spaGFM can transfer learned spatial representations across independent datasets, cancer cohorts, and tissue-processing protocols.

#### CRISPR screening and spatial transcriptomics of Mouse Xenograft models in Perturb-FISH

To benchmark spatially-resolved genetic-perturbation prediction, we used the perturb-FISH in mouse tumor dataset, downloaded from the Brain Image Library (https://download.brainimagelibrary.org/0c/bd/0cbd479c521afff9/extras/tumors/processed/finaltables/). For each sample, we assembled an *AnnData* (.h5ad) object from the two provided final tables: merfishcounttable.csv, the MERFISH gene-by-cell count matrix, and coordinates.csv, the spatial centroid (x, y) of every segmented cell. The two tables were joined on cell identifier so that each cell’s expression profile was paired with its spatial coordinate, and tumor versus T-cell identity labels together with the CRISPR perturbation (target gene) assigned to each cell were read from the matching records of the count table by cell-number matching. Because the original perturb-FISH analysis derives each cell’s microenvironment from segmented cell boundaries (membrane-to-membrane contact), whereas the public tables provide only cell centroids and not boundary polygons, we reconstructed the neighborhood graph from centroids and benchmarked several approximation strategies against the boundary-derived reference, including k-nearest-neighbors, nearest-cell classification, a preliminary distance-based classifier, and a size-aware classifier, across a range of parameters. A k-nearest-neighbor graph with k=4 most closely reproduced the original boundary-based neighbor relationships and was therefore adopted for all downstream spatial analyses. Ground-truth perturbation effects (log-fold changes) were computed with the FR-Perturb framework using the FR-PerturbnoNorm.py script from the Perturb-Fish GitHub repository.

#### Human diabetic kidney disease (DKD) studies in 10x Xenium spatial transcriptomics

Spatially resolved single-cell transcriptomic profiling of a human diabetic kidney disease specimen (sample ID IU04) was generated on the 10x Genomics Xenium platform^39^. The severity of glomerular injury was manually annotated by biologists with nephropathologist expertise.

##### Tissue and assay.

A formalin-fixed paraffin-embedded (FFPE) human kidney section was assayed on the Xenium Analyzer (instrument XETG00126, slide 0015383) using the Xenium chemistry v1 with the Xenium Multi-Tissue segmentation stain, and decoded with Xenium Ranger analysis software (v3.2.0). Transcripts were imaged across a tissue region of ≈34.3 mm^2^. A total of 15,786,934 transcripts were detected, of which 13,172,460 (83%) were high-quality decoded transcripts and 78.3% were assigned to cells.

##### Gene panel.

Detection used a custom 300-plex human kidney panel (hKidney_300g, panel design BMU84Y, organism *Homo sapiens*, tissue type Kidney). The feature matrix retains all 541 codewords reported by Xenium (300 Gene Expression targets, 20 Negative Control Probes, 41 Negative Control Codewords, and 180 Unassigned Codewords), enabling background/false-discovery estimation. Only the 300 Gene Expression features should be used for downstream biological analysis. Negative-control signal was negligible (216 negative-control-probe and 0 genomic-control counts across all cells).

##### Cells and segmentation.

After cell segmentation, the dataset comprises 84,647 cells by 541 features (300 genes). Cells were segmented primarily by interior 18S stain (73,402 cells, 86.7%), supplemented by boundary stain (ATP1A1+CD45+E-Cadherin; 6,290 cells, 7.4%) and 5.0-μm nuclear-expansion segmentation (4,955 cells, 5.9%). On average, cells contained a median of 89 transcripts (mean 121.8) spanning a median of 41 detected genes (mean 41.7), with a median cell area of $55.3\;\mu {\text{m}}^{2}$.

### Evaluation settings for downstream tasks

#### Benchmarking embedding representations in Human and Mouse datasets

Each CosMx human and mouse tissue slice was evaluated independently to assess cross-graph inductive transfer under a transductive target-graph setting, in which the frozen pretrained encoder generated cell embeddings using the full topology of each target graph. This design was chosen because niche or cell type annotations can be sample-specific and may not correspond one-to-one across patients. For zero-shot evaluation, the pretrained model was frozen and applied directly to each held-out dataset to generate cell embeddings. For linear probing, a linear classifier was trained to predict niche labels for 100 epochs using a learning rate of 0.01 and a weight decay of 0.001. For k-nearest-neighbor (k-NN) evaluation, we used k equals 5.

For both supervised evaluation settings, cells within each slice were randomly partitioned into training, validation, and test sets at ratios of 0.1, 0.1, and 0.8, respectively. Label stratification was not enforced, reflecting a generic low-label sampling setting. This procedure was repeated ten times using independent random seeds to account for sampling variability. For unsupervised evaluation, k-means clustering was performed on the full set of cell embeddings from each slice in ten independent runs with different random seeds. Final performance metrics were reported as the mean across the ten runs.

For the Xenium human lung and brain datasets, in which niche annotations were shared across samples, we additionally evaluated cross-sample label transfer. Samples were randomly partitioned into training and test sets at a ratio of 0.2:0.8. Cell embeddings from the training samples were used as the reference set for k-NN-based label transfer to cells in the held-out test samples, with k=5. This sample-level split ensured that cells from each test sample were excluded from the reference set used for label transfer. The evaluation was repeated ten times using independent random seeds, and performance was summarized as the mean across runs.

For comparison with existing methods, we followed the recommended settings for each benchmark model and extracted zero-shot cell representations from Novae, Nicheformer, and scGPT-spatial. We used the human-pretrained Novae model for the CosMx human datasets and the mouse-pretrained Novae model for the CosMx mouse brain datasets. The resulting representations were evaluated using the same downstream protocols as those applied to spaGFM.

#### Simulation of graph corruption and cell segmentation artifacts in CosMx Human Lung

##### Topological noises.

We simulated two types of noise for graph topology: node feature shuffling and node position shifting. For each slice i, we constructed spatial cellular graphs with corrupted nodes at four proportions: {0, 0.1, 0.3, 0.5}. In the node feature shuffling setting, node features were randomly permuted according to the specified corruption ratios. In the node position-shifting setting, candidate node coordinates were perturbed using offsets sampled from a Gaussian distribution with mean 0 and standard deviation 10, scaled by the centroid distance to their k-nearest neighbors (k=30).

##### Geometric boundary perturbations.

To evaluate sensitivity to cell-boundary geometry in CosMx data, we perturb each cell boundary polygon independently with a randomized affine transformation whose magnitude is controlled by a single intensity scalar λ. Let a cell i located at coordinates (x,y) be represented by its boundary vertices ${\left\{p_{i}=\left(x_{i},y_{i}\right)\right\}}_{i=1}^{N}$ with centroid $c=\frac{1}{N}\sum_{i}p_{i}$. We first center the polygon, ${\tilde{p}}_{i}=p_{i}-c$, and then apply a sequence of a rotation, an isotropic scaling, an anisotropic shear, and a translation. The transformation parameters are drawn independently per cell from uniform distributions whose ranges scale linearly with λ:

$$\theta \sim 𝒰(-15\lambda ,15\lambda {)}^{\circ },s\sim 𝒰(1-0.1\lambda ,1+0.1\lambda ),$$

$$\phi_{x},\phi_{y}\sim 𝒰(-5\lambda ,5\lambda {)}^{\circ },t_{x},t_{y}\sim 𝒰(-10\lambda ,10\lambda ),$$

Here, U(a,b) denotes a uniform distribution over the interval [a,b],θ is the rotation angle, s is the isotropic scaling factor applied equally to both spatial dimensions, $\phi_{x}$ and $\phi_{y}$ are the shear angles along the x - and y-directions, respectively, and $t_{x},t_{y}$ is the translation vector along the two spatial axes. The parameter λ jointly governs the maximum rotation (15°), area scaling (±10%), shear (5°), and translation (10 px) at λ=1 (we use λ=0.5 by default). Each centered vertex is first rotated,

x′y′=cosθ−sinθsinθcosθx~y~,

then uniformly scaled with isotropic scaling factor $s,\left(x^{\prime \prime },y^{\prime \prime }\right)=\left(sx^{\prime },sy^{\prime }\right)$, and sheared sequentially along x and y:

$$x^{\prime \prime \prime }=x^{\prime \prime }+\text{tan}\left(\phi_{x}\right)y^{\prime \prime },y^{\prime \prime \prime }=y^{\prime \prime }+\text{tan}\left(\phi_{y}\right)x^{\prime \prime \prime }.$$

Note that the y-shear is applied to the already x-sheared coordinate $x^{\prime \prime \prime }$, giving a mildly non-orthogonal composite shear. Finally, the polygon is re-centered and translated, $p_{i}^{\text{new}}=\left(x^{\prime \prime \prime },y^{\prime \prime \prime }\right)+c+\left(t_{x},t_{y}\right)$, and vertices are rounded to integer pixel coordinates.

To preserve physically plausible cell segmentations, we enforce non-overlap through rejection sampling. For each cell, up to K candidate transformations (default K=30) were generated, and the first candidate that produced a geometrically valid polygon (repaired using a zero-width buffer operation where necessary) without intersecting any previously accepted cell was retained. Cells with fewer than four vertices or topologically invalid boundaries were excluded from perturbation. This procedure generated controlled, spatially coherent geometric perturbations while maintaining a valid, non-overlapping tessellation of the tissue.

#### The identification of Tertiary Lymphoid Structures (TLS) in 10x Visium Human Lung and Kidney

To predict TLS regions, we employed a randomly initialized attention-based TLS decoder, (D(⋅)), which transformed each neighborhood representation $h_{ij}$ from Eq. (2) into a TLS prediction ${\overset{ˆ}{y}}_{ij}^{\text{TLS}}=D\left(h_{ij}\right)$. The decoder and spaGFM parameters were jointly optimized using the cross-entropy loss $ℒ=\text{CrossEntropy}\left(y_{ij}^{\text{TLS}},{\overset{ˆ}{y}}_{ij}^{\text{TLS}}\right)$, where $y_{ij}^{\text{TLS}}$ denotes the annotated TLS annotation. Model training was performed using the Adam optimizer with a learning rate of 0.01 and weight decay of 0.001 for 50 epochs. Spots lacking TLS annotations were excluded from both training and evaluation. We compared spaGFM with Scanpy, NicheCompass, Novae, scGPT-spatial, native scGPT and fine-tuned scGPT. For these competitive methods, TLS regions were identified using the corresponding label-transfer workflows. Briefly, embeddings of query spots were compared with embeddings of annotated reference spots, and the k-nearest neighbors (K=10, following the scGPT study) were identified in the embedding space. The TLS label for each query spot was assigned by majority vote among its nearest annotated neighbors. Given the class imbalance among early-stage TLS, mature TLS, and non-TLS spots, model performance was assessed using the F1-macro score. In addition, we calculated a continuous TLS signature score for each spot as the mean log-normalized expression of 21 canonical TLS marker genes (*MS4A1, CD79A, CD79B, MZB1, IGHG1, CCL19, CCL21, CCR7, SELL, LAMP3, CR2, BCL6, CXCL13, CXCR5, CD3D, CD3E, CD8A, PDCD1* and *CHST4, CHST2, and MADCAM1*)^18,26,47,48^, capturing coordinated B-cell, T-cell and follicular chemokine programs associated with TLS formation. To assess whether predicted TLS regions formed spatially coherent structures rather than dispersed predictions, we constructed a spatial neighborhood graph by connecting each spot to its six nearest neighbors. Spatial organization was quantified using Moran’s I, a global measure of spatial autocorrelation computed on predicted or annotated TLS labels^49,50^. Moran’s I ranges from approximately −1 (spatial dispersion) to +1 (strong spatial clustering), with values near zero indicating spatial randomness. As a complementary metric, we calculated neighborhood purity^51,52^ as the fraction of the six nearest neighbors sharing the same TLS label as the focal spot and reported the average purity across all spots. Neighborhood purity quantifies the local consistency of TLS predictions, with a value of 1 indicating perfectly homogeneous neighborhoods. Accordingly, this analysis compares method-specific prediction pipelines rather than frozen representations under a common downstream classifier.

#### Prediction of spatially conditioned perturbation responses in Perturb-FISH mouse xenografts

We analyzed a spatial CRISPR perturbation atlas generated by perturb-FISH, an imaging-based spatial transcriptomics assay. The dataset comprises approximately 153,000 cells profiled with a 500-gene MERFISH panel. Each cell is annotated with a single-gene knockout (KO) or non-targeting control label, two-dimensional spatial coordinates, and a 500-dimensional gene expression profile.

For every perturbed cell, the effect of each panel gene was quantified as the log-fold change (LFC) relative to identity-matched control cells. LFC values were estimated using the Factorize-Recover framework (FR-Perturb) following the original Perturb-FISH study and served as supervision targets throughout model training. GPT-3.5 gene embeddings were obtained from GenePT^28^ for every perturbation gene and kept fixed across all experiments.

To operationally define T-cell neighborhood status, we evaluated candidate spatial definitions according to whether adding the resulting binary neighbor indicator improved prediction of perturbation-induced expression classes beyond perturbation identity alone. For each candidate definition, tumor cells were partitioned into neighbor-positive and neighbor-negative groups, perturbation-specific LFC profiles were re-estimated separately for the two groups, and gene-level LFCs were discretized as downregulated, unchanged, or upregulated using margins of 0.05, 0.10, 0.15, and 0.20. Ridge classifiers were then fit using either the compressed GenePT perturbation embedding alone or the GenePT embedding together with the binary neighbor indicator. Based on this analysis, local T-cell proximity was defined as the mean Euclidean distance to the four nearest T cells. Tumor cells at or below the 55th percentile of this distribution were classified as T-cell-proximal (with T-cell neighbors), whereas the remaining cells were classified as T-cell-distal (without T-cell neighbors). This binary label was used to define T-cell context and was not used as an input to the downstream spaGFM perturbation-prediction model. The tissue graph was constructed as described in the “Spatial cellular graph construction” section. A pretrained spaGFM model generated a 1,280-dimensional cell embedding that served as the spatial input for subsequent analyses.

Perturbation prediction was formulated as a binary classification task. For every perturbed cell and panel gene, the model predicted whether the corresponding LFC was positive or negative. The predictor comprised two multilayer perceptron branches. The gene branch encoded the fixed GenePT embedding, whereas the spatial branch received the spaGFM embedding. Both branches projected their inputs into a shared 256-dimensional latent space through a linear layer followed by ReLU activation and dropout. Their outputs were concatenated and passed through a multilayer fusion network to produce a single logit, which was converted into a probability using a sigmoid function. Prediction confidence for each perturbation, gene, and T-cell context was summarized by averaging sigmoid probabilities across cells. Scores were linearly rescaled from [0,1] to [−1,1]. The sign denotes the predicted regulation direction, whereas the magnitude reflects classification confidence. For the effect-size-stratified analysis in Supplementary Fig. 15g, this signed confidence score was compared with observed LFC profiles using Spearman correlation; this statistic assesses confidence-to-LFC concordance rather than output from a separate effect-size regression model.

All methods were optimized with class-weighted binary cross-entropy loss. Gene-specific positive weights compensated for class imbalance. Optimization employed AdamW with a learning rate of 1 × 10^−3^) and a weight decay of 1 × 10^−4^. Models were trained with a batch size of 256 for up to 200 epochs. Early stopping was monitored on a validation set containing 10% of the training data, with a patience of 20 epochs. A fixed random seed of 42 was used throughout. The embedding-based comparators shared an identical architecture and optimization schedule and differed only in the spatial input. spaGFM received pretrained spaGFM embeddings. The MLP baseline replaced the spatial input with an all-zero vector. Celcomen served as a spatial counterfactual comparator. It generates perturbation effects natively and shares neither the architecture nor the optimization schedule of the embedding-based arms. Fitted on the same tissue graph, it produced a counterfactual by setting the target gene to its knockout state in the perturbed cell, and the predicted direction was taken as the sign of the counterfactual-minus-observed difference. GEARS served as a non-spatial baseline. It was trained for 20 epochs with a hidden dimension of 64. Predicted perturbation effects were converted to LFC values relative to the local control mean and assigned to all cells belonging to the corresponding perturbation.

Performance was evaluated using ten predefined leave-perturbation-out splits, with a minimum of 20 test cells required for each split-perturbation pair. This yielded 70 evaluable pairs for spaGFM and GenePT. For the direct four-method comparison, we further retained only perturbations whose target genes were represented in the measured panel and were therefore jointly evaluable by GEARS and Celcomen, resulting in 10 eligible perturbations and 24 common split-perturbation pairs. Fig. 4d reports all four methods on exactly these 24 pairs, whereas analyses involving only spaGFM and GenePT retained all 70 pairs.

The T-cell spatial context was evaluated by predicting the binary T-cell proximity label from cell embeddings using logistic regression under five-fold stratified cross-validation. Spatially driven heterogeneity was quantified using residual LFC, defined as the difference between the observed LFC of a cell and the mean LFC of its perturbation. Ridge regression was used to predict residual LFC for the 50 genes with the largest residual variance among the 11 perturbations containing at least 20 cells. Prediction followed leave-one-perturbation-out cross-validation. Three feature sets were compared. The first comprised handcrafted T-cell spatial descriptors, including nearest-neighbor identity, k-nearest-neighbor composition, cell-size-aware neighborhood membership, and the number of neighboring T cells. The second comprised PCA-reduced scGPT niche embeddings computed from non-T cells within a radius of 300 spatial units. The third comprised PCA-reduced spaGFM embeddings generated with a walk length of nine.

In attention analysis, reach was defined for each focal perturbed tumor cell as the attention-weighted mean Euclidean distance to its walk-reachable neighboring cells. Cliff’s delta was calculated as δ=P(X>Y)−P(X<Y), where X and Y denote attention-reach values for T-cell-proximal and T-cell-distal tumor cells, respectively. Statistical significance was assessed using two-sided paired Wilcoxon signed-rank tests by comparing spaGFM with each competing method across matched perturbation-split pairs. Cross-validation AUC and R^2^ are reported as mean ± standard deviation across folds.

#### The characterization of glomerulus organizations in 10x Xenium Human Kidney

##### Dataset and pathological annotation.

We analyzed a kidney biopsy from a DKD patient within the KPMP atlas. The sample comprised four serial sections (Splits 1–4) profiled by SRT on the 10x Genomics Xenium platform. For each section, cell-type identities and glomerular boundaries were manually annotated. A pathologist graded glomerular pathological severity from H&E morphology into four categories: no evident glomerular pathology (Grade 0), mild (Grade 1), moderate (Grade 2), and severe (Grade 3). Cell embeddings were generated with spaGFM as described before. For the zero-shot analyses, embeddings were used directly from the pretrained model without any task-specific training on the kidney data.

##### Zero-shot glomerulus identification.

To assess whether spaGFM resolves glomerular microstructure without supervision, we performed Leiden clustering (resolution = 0.5) on the Novae zero-shot cell embeddings, yielding 11 spatial domains. We then applied Leiden clustering to the spaGFM zero-shot cell embeddings, adjusting the resolution parameter to obtain the same number of clusters for comparison.

##### Trajectory and pseudo-time analysis.

Glomerular cells were extracted by manual annotations, and their embeddings were embedded in two dimensions with UMAP and clustered. Pseudo-time trajectories were inferred with Slingshot^53^ over the UMAP representation.

##### Glomerulus and disease-state classification.

For supervised detection, we attached an MLP classifier to the model and fine-tuned on 13 annotated glomeruli from Split 2, validated on five glomeruli from Split 4, and tested on the remaining two sections. Two tasks were performed: (i) per-cell binary classification of glomerulus versus non-glomerulus, and (ii) Grade 0 versus Grades 1–3 glomerulus classification, for which Grades 1 to 3 were merged into a single diseased category. Performance was quantified by accuracy, precision, recall, and F1 score. Fine-tuning settings are detailed in the “Fine-tuning” section.

##### Attention-weight analysis.

For each attention head, we represented its attention pattern as a two-dimensional vector field $F=\left(F_{x},F_{y}\right)$ over the spatial coordinates of the tissue and treated this field as a spatial flow. Each vector encodes both the direction and magnitude of attention flow between cells. The vector direction indicates the dominant spatial orientation along which attention is propagated, whereas the vector magnitude reflects the strength of the corresponding attention flow. We characterized its local geometry with two standard differential operators. The curl,

$$\nabla \times F=\frac{\partial F_{y}}{\partial x}-\frac{\partial F_{x}}{\partial y},$$

measures the local rotational structure of the flow, and the divergence,

$$\nabla \cdot F=\frac{\partial F_{x}}{\partial x}+\frac{\partial F_{y}}{\partial y},$$

measures local expansion or contraction, with positive values indicating a source (attention spreading outward) and negative values a sink (attention concentrating inward). For each glomerulus, the spatial derivatives of the vector components were jointly estimated from the attention vector and coordinate differences among cells within that glomerulus using least-squares fitting of the spatial gradients. The resulting derivatives were used to calculate glomerulus-level divergence and absolute curl. Statistical comparisons across grades were performed using two-sided Mann-Whitney U tests.

## Supplementary Files

This is a list of supplementary files associated with this preprint. Click to download.

- supplfiles.docx

## Data Availability

Pre-training datasets are publicly available from the corresponding official repositories: Xenium (https://www.10xgenomics.com/datasets), CosMx (https://brukerspatialbiology.com/products/cosmx-spatial-molecular-imager/ffpe-dataset) and MERSCOPE (https://vizgen.com/data-release-program). Sample-level metadata is available at Supplementary Data 1. The HuBMAP reference or diseased tissue data can be accessed through the HuBMAP publication page upon acceptance of the peer-reviewed manuscript. Source data are provided with this paper.

## Code availability

The official repository of spaGFM is available at GitHub (https://github.com/yzhong36/spaGFM). The reproducibility repository of spaGFM is available at GitHub (https://github.com/yzhong36/spaGFM_reproducibility).
